# Universal cutoff for tumor mutational burden in predicting the efficacy of anti-PD-(L)1 therapy for advanced cancers

**DOI:** 10.3389/fcell.2023.1209243

**Published:** 2023-05-25

**Authors:** Shu-Fen Mo, Zeng-Zhi Cai, Wen-Hao Kuai, Xuexin Li, Yu-Tong Chen

**Affiliations:** ^1^ Department of Medical Oncology, Guangdong Agriculture Reclamation Central Hospital, Zhanjiang, China; ^2^ Department of Dermatology, Changhai Hospital of Shanghai, Second Military Medical University (Naval Medical University), Shanghai, China; ^3^ Department of Medical Biochemistry and Biophysics, Karolinska Institute, Stockholm, Sweden; ^4^ Faculty of Medical Science, Jinan University, Guangzhou, China

**Keywords:** tumor mutational burden (TMB), anti-PD-(L)1 therapy, advanced solid tumors, universal cutoff, biomarker

## Abstract

**Background:** The US Food and Drug Administration (FDA)’s tumor-agnostic approval of pembrolizumab in high tumor mutational burden (TMB-high, i.e., TMB≥10 mut/Mb) cases, based on the data from KEYNOTE-158, has raised considerable concerns among the immuno-oncology community. This study aims to statistically infer the optimal universal cutoff in defining TMB-high that is predictive of the efficacy of anti-PD-(L) 1 therapy in advanced solid tumors.

**Methods:** We integrated MSK-IMPACT TMB data from a public cohort and the objective response rate (ORR) for anti-PD-(L) 1 monotherapy across diverse cancer types in published trials. The optimal TMB cutoff was determined by varying the universal cutoff to define TMB-high across cancer types and examining the cancer-level correlation between objective response rate and the proportion of TMB-high cases. The utility of this cutoff in predicting overall survival (OS) benefits from anti-PD-(L) 1 therapy was then evaluated in a validation cohort of advanced cancers with coupled MSK-IMPACT TMB and OS data. In silico analysis of whole-exome sequencing data from The Cancer Genome Atlas was further employed to assess the generalizability of the identified cutoff among panels comprising several hundred genes.

**Results:** The cancer type-level analysis identified 10 mut/Mb as the optimal cutoff for MSK-IMPACT in defining TMB-high, with the corresponding TMB-high (TMB≥10 mut/Mb) percentage strongly correlated with ORR for PD-(L) 1 blockade across cancer types [correlation coefficient, 0.72 (95% CI, 0.45–0.88)]. This cutoff was also the optimum in defining TMB-high (via MSK-IMPACT) when predicting OS benefits from anti-PD-(L) 1 therapy in the validation cohort. In this cohort, TMB≥10 mut/Mb was associated with significantly improved OS (hazard ratio, 0.58 [95% CI, 0.48–0.71]; *p* < 0.001). Moreover, *in silico* analyses revealed excellent agreement of TMB≥10 mut/Mb cases between MSK-IMPACT and the FDA-approved panels and between MSK-IMPACT and various randomly sampled panels.

**Conclusion:** Our study demonstrates that 10 mut/Mb is the optimal, universal cutoff for TMB-high that guides the clinical application of anti-PD-(L) 1 therapy for advanced solid tumors. It also provides rigorous evidence beyond KEYNOTE-158 for the utility of TMB≥10 mut/Mb in predicting the efficacy of PD-(L) 1 blockade in broader settings, which could help to mitigate the challenges in embracing the tumor-agnostic approval of pembrolizumab in TMB-high cases.

## Introduction

Anti-PD-(L) 1 therapy has achieved great success in more than 15 cancer types, given its prolonged duration of response and favorable tolerability profile (Wang et al., 2021; Sharma et al., 2023). However, only a minor subset of patients benefit from this treatment and clinical responses vary greatly across cancer types (Wang et al., 2023). As such, there is a crucial need of robust biomarkers in predicting the efficacy of PD-(L)1 inhibitors to guide patient selection for such treatment (Hegde and Chen, 2020; Wang and Xu, 2023).

Tumor and/or immune cell PD-L1 expression by immunohistochemical assays was first approved by the US Food and Drug Administration (FDA) as a companion diagnostic to pembrolizumab for advanced non-small-cell lung cancer, upper gastrointestinal cancers, cervical cancer, and urothelial cancer, based on convincing evidence for these malignancies (Herbst et al., 2016; Muro et al., 2016; Reck et al., 2016; Bellmunt et al., 2017; Chung et al., 2018; Fuchs et al., 2018). However, this marker alone is insufficient for the precise prediction of anti-PD-(L) 1 response, and there is a lack of standardization across platforms, scoring systems and cutoff values for PD-L1 expression (Wang and Xu, 2023). In addition, high microsatellite instability (MSI-H), an indicator of defects in DNA repair that gives rise to a large quantity of neoantigens, was approved by the FDA in a tumor-agnostic manner, but it was largely aggregated in several malignancies (e.g., colorectal cancer, gastric cancer, and endometrial cancer), with an overall low prevalence (Le et al., 2015; Hause et al., 2016; Diaz et al., 2017; Overman et al., 2017).

Tumor mutational burden (TMB), generally referred to as the total number of mutational events in the genome of a tumor and reflective of tumor immunogenicity, is emerging as a promising biomarker for PD-(L) 1 blockade (Chan et al., 2019). Although whole-exome sequencing (WES) is the standard approach for measuring TMB, targeted next-generation sequencing (NGS) panels appear to be more pragmatic for TMB estimation, given that routine implementation of WES in clinical practice is limited by its substantial costs and long turnaround times (Wu et al., 2019; Merino et al., 2020). Since 2015, mounting evidence has shown that high TMB is associated with greater clinical benefits from anti-PD-(L) 1 therapy among various malignancies (Rizvi et al., 2015; Samstein et al., 2019; Singal et al., 2019; Wang et al., 2023). Most recently, the KEYNOTE-158 study revealed an objective response rate (ORR) of 29% with pembrolizumab for treating refractory advanced solid tumors with high TMB, i.e., TMB≥10 mutations per megabase (mut/Mb) as determined by FoundationOne CDx (F1CDx) (Marabelle et al., 2019; Marabelle et al., 2020); thereby, TMB-high was recently approved by the FDA as another tumor-agnostic companion diagnostic to pembrolizumab in patients refractory to standard treatments (FDA approves, 2020).

Nevertheless, this tumor-agnostic approval based on KEYNOTE-158 has raised considerable concerns and doubts among the immuno-oncology community (Bersanelli, 2020; Prasad and Addeo, 2020). Firstly, the cutoff of 10 mut/Mb in defining TMB-high was not statistically inferred based on anti-PD-(L) 1 efficacy data. Secondly, significant overall survival (OS) benefits in TMB-high cases were not demonstrated with this cutoff. Last but not least, it remains unknown whether this cutoff can be extrapolated to other tumor types, NGS panels, and PD-(L) 1 inhibitors that were not involved in KEYNOTE-158.

Despite these limitations, KEYNOTE-158 has provided promising evidence for the utility of a universal TMB cutoff across cancer types for prediction of anti-PD-(L) 1 response, which is of particular value as it would be challenging and labor-intensive to establish tumor-specific TMB thresholds. In this study, we integrated large-scale genomic and clinical data and performed cancer type-level and patient-level analyses to statistically infer the optimal, universal cutoff in defining TMB-high and predicting the efficacy of anti-PD-(L) 1 therapy in advanced solid tumors.

## Materials and methods

### Study cohort and study design

**FIGURE 1:**
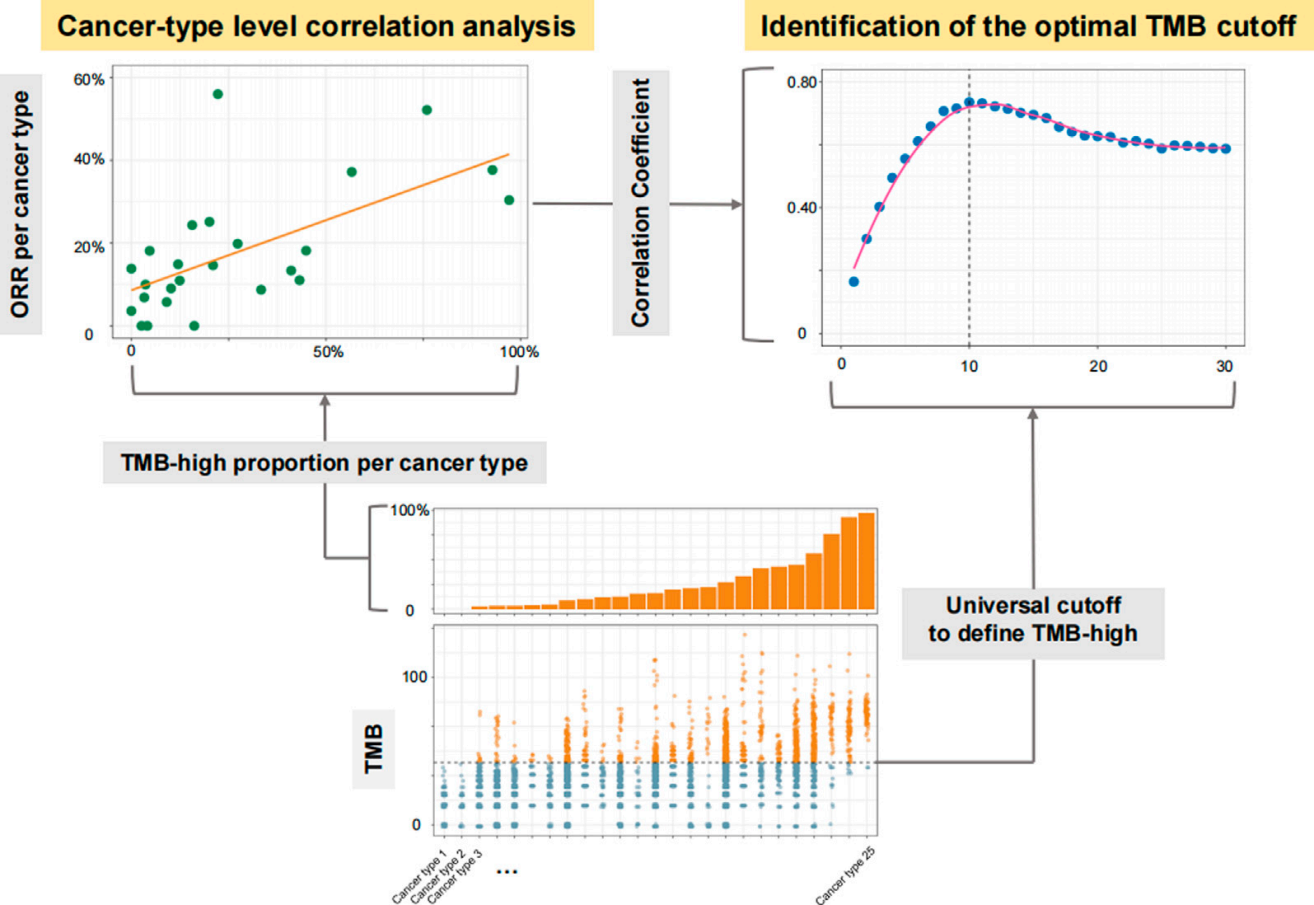
Diagram for the process of identifying the optimal universal cutoff for tumor mutational burden (TMB) in predicting responses to PD-(L) 1 inhibitors. For each given universal TMB cutoff, the proportion of TMB-high cases defined by this cutoff was calculated for each cancer type. The cancer type-level correlation between objective response rate (ORR) and the proportion of TMB-high cases was then evaluated. By varying the universal cutoff to define TMB-high, the curve for the association between such cancer type-level correlation and the universal TMB cutoff was depicted, and the Chow test was then applied to determine the structural breakpoint, hence the optimal TMB cutoff.

**FIGURE 2:**
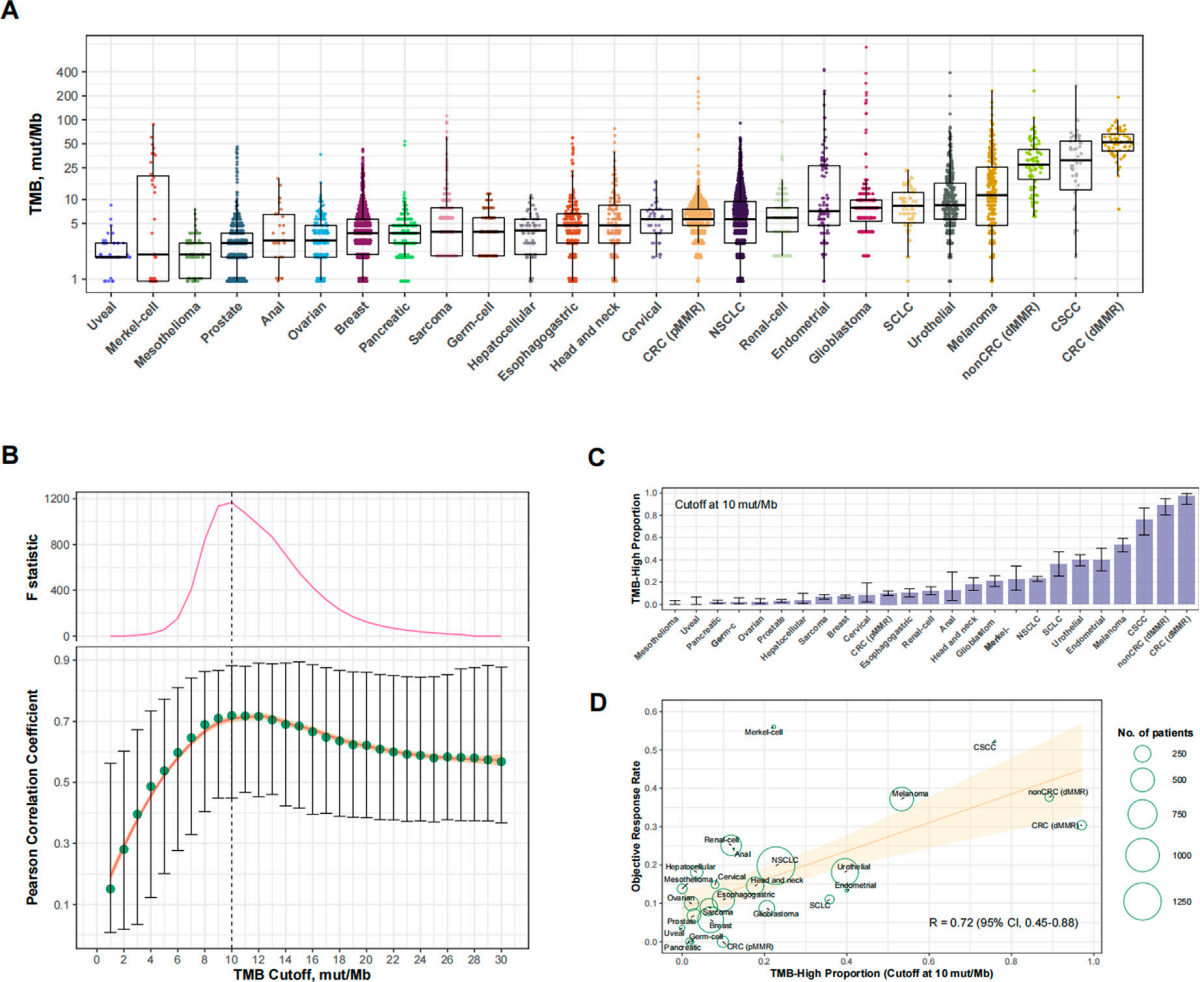
Cancer type-level analysis to identify the optimal universal cutoff for tumor mutational burden (TMB) in predicting responses to PD-(L)1 blockade. **(A)** The landscape of TMB across the 25 eligible cancer types in Cohort 1. Abbreviations: NSCLC, non-small-cell lung cancer; CRC (dMMR), microsatellite-instable colorectal cancer; CRC (pMMR), microsatellite-stable colorectal cancer; nonCRC (dMMR), microsatellite-instable non-colorectal cancer; CSCC, cutaneous squamous-cell carcinoma. **(B)** Cancer type-level correlation between anti-PD-(L)1 response rate and the proportion of TMB-high cases, as well as the Chow F statistic, are plotted against varying cutoff for TMB. The yellow curve denotes the LOESS smoother. A structural breakpoint at 10 that maximized the Chow F statistic (i.e., minimizing the ordinary least squares estimator) was identified by Chow test. **(C)** The proportion of TMB-high (i.e., TMB≥10 mut/Mb). cases across cancer types in Cohort 1. **(D)** Cancer type-level correlation between anti-PD-(L) 1 response rate and the proportion of TMB-high when the cutoff was 10 mut/Mb.

This study was performed from 27 July 2020, to 15 September 2020. Three steps were included: 1) Cancer type-level analysis to identify the optimal TMB cutoff in predicting responses to PD-(L) 1 inhibitors (Figure 1); 2) patient-level analysis to verify the utility of TMB-high defined by the identified cutoff in predicting the efficacy of PD-(L) 1 inhibitors; and 3) *in silico* analysis to assess the generalizability of the identified TMB cutoff among existing targeted sequencing panels and randomly sampled panels comprising several hundred genes.

At Step 1, we included cancer types with available MSK-IMPACT TMB data as well as anti-PD-(L) 1 monotherapy ORR data consolidated by Yarchoan et al. (Wang et al., 2023) The MSK-IMPACT TMB data were obtained from a public cohort involving over 10,000 advanced cancer patients (Cohort 1) (Zehir et al., 2017). Only cancer types with at least 30 cases in Cohort 1 and at least 10 participants in trials investigating anti-PD-(L) 1 monotherapy were included. The optimal TMB cutoff was determined by varying the universal cutoff to define TMB-high across cancer types and examining the corresponding cancer-level association between ORR and the proportion of TMB-high cases (Figure 1).

At Step 2, we analyzed a public cohort involving over 1,000 advanced cancer patients receiving anti-PD-(L) 1 monotherapy with coupled MSK-IMPACT TMB data and overall survival (OS) data (Cohort 2) (Samstein et al., 2019). We noticed some overlapped patients in Cohorts 1 and 2, but they were not excluded from the analyses at Step 2 as the TMB and ORR data at Step 1 were disconnected, i.e., from different populations. TMB-high was defined by the cutoff identified in Step 1, and the association between TMB status (high vs. low) and OS was evaluated.

At Step 3, we analyzed WES data from The Cancer Genome Atlas (TCGA) across 32 solid tumors. The MC3 somatic mutation data were downloaded from the UCSC Xena browser (http://xena.ucsc.edu/). When data on more than one tumor sample were available, the primary tumor sample was the favored choice, and in remaining cases “metastatic” was selected over “additional metastatic”. We first generated *in silico* gene panels that comprised genes from the three NGS panels that have thus far received FDA approval or authorization (i.e., MSK-IMPACT, F1CDx, and PGDx elio tissue complete [PGDx]) (Chalmers et al., 2017; Zehir et al., 2017; Wood et al., 2018). TMB for each *in silico* panel was calculated using its own unique bioinformatics pipeline. We also generated *in silico* panels with various sizes and percentages of shared genes with MSK-IMPACT, with 1,000 resampling for each given panel size and percentage of shared genes. We performed three cycles of analyses of randomly sampled panels; in each cycle, we applied one of the bioinformatics pipelines of the three FDA-approved or authorized panels to calculate TMB for the randomly sampled panels. We then used the cutoff identified in Step 1 to define TMB-high across all these panels, and evaluated their concordance with MSK-IMPACT in defining TMB-high cases.

Detailed patient/sample inclusion and exclusion procedures are shown in Supplementary Figure S1

### Statistical analysis

At Step 1, linear regression models were fitted by use of ordinary least-squares regression to examine the cancer-level associations between ORR and the proportion of TMB-high cases. The linear models were weighted by the geometric means of the sample size of trials and that of Cohort 1 per cancer type. The strength of association was indicated by the square root (*R*_*TMB-ORR*_) of the coefficient of determination from the linear models. We then depicted the curve for the association between *R*_*TMB-ORR*_ and the universal cutoff to define TMB-high across cancer types, and applied the Chow test to determine the structural breakpoint (Zeileis et al., 2010). The structural breakpoint was considered as the threshold of clinico-biological impact, hence the optimal TMB cutoff for further investigation.

At Step 2, hazard ratios (HRs) and corresponding 95% confidence intervals (CIs) for the association between TMB status (high vs. low) and OS was evaluated using Cox proportional hazards models adjusted for age, sex, MSK-IMPACT version, and cancer type. We depicted the curve for the association between the Wald test Z score (i.e., β coefficient of TMB divided by its standard error in the multivariable Cox proportional hazards model) and the universal cutoff to define TMB-high across cancer types, and applied the Chow test to determine the structural breakpoint. Concordance indices (C-indices) were used to assess the discriminatory capacity of models (Pencina and D'Agostino, 2004). In this step, the 80th percentile was used as the tumor-specific TMB cutoff to be compared with the universal cutoff of 10 mut/Mb; the 80th percentile was chosen because it was identified as the optimal percentile cutoff to predict the efficacy of PD-(L) 1 blockade in Samstein’s study (Samstein et al., 2019).

At Step 3, the Cohen’s Kappa coefficient, a statistic measuring inter-rater reliability, was used to evaluate the agreement between TMB-high cases based on MSK-IMPACT and *in silico* panels using the same cutoff. A Kappa of 1 indicates perfect agreement and a Kappa above 0.80 indicates an almost perfect agreement (McHugh, 2012).

The significance level was set at a two-sided *p* < 0.05. All statistical analyses were performed using R software version 3.6.1 (http://www.r-project.org).

### Role of the funding source

The funder of this study had no role in its design, data collection, data analysis, data interpretation, and writing of the report. The corresponding author had full access to all the data in the study and had final responsibility for the decision to submit for publication.

## Results

### Optimal TMB cutoff in predicting responses to PD-(L)1 blockade at the cancer type level

We analyzed a total of 25 eligible cancer types with at least 30 cases in Cohort 1 (totaling 8,201 cases) and at least 10 participants in trials investigating anti-PD-(L) 1 monotherapy (totaling 6,348 cases; Supplementary Table S1). The ORR ranged from 0% to 56.0% across cancer types. The level of TMB varied greatly across cancer types (Figure 2A). Figure 2B shows the relationship between the TMB cutoff and the corresponding *R*_*TMB-ORR*_. With a higher cutoff for TMB, the *R*_*TMB-ORR*_ increased and then plateaued, and a structural breakpoint of 10 mut/Mb was identified by the Chow test (Figure 2B). When this cutoff was used to define TMB-high (TMB≥10 mut/Mb), the proportion of TMB-high cases ranged from 0% to 97.1% across cancer types (totaling 16.2% for the entire cohort; Figure 2C), with *R*_*TMB-ORR*_ reaching 0.72 (95% CI, 0.45–0.88; *p* < 0.001) (Figure 2D).

### Verification of the reliability of the identified TMB cutoff

**FIGURE 3:**
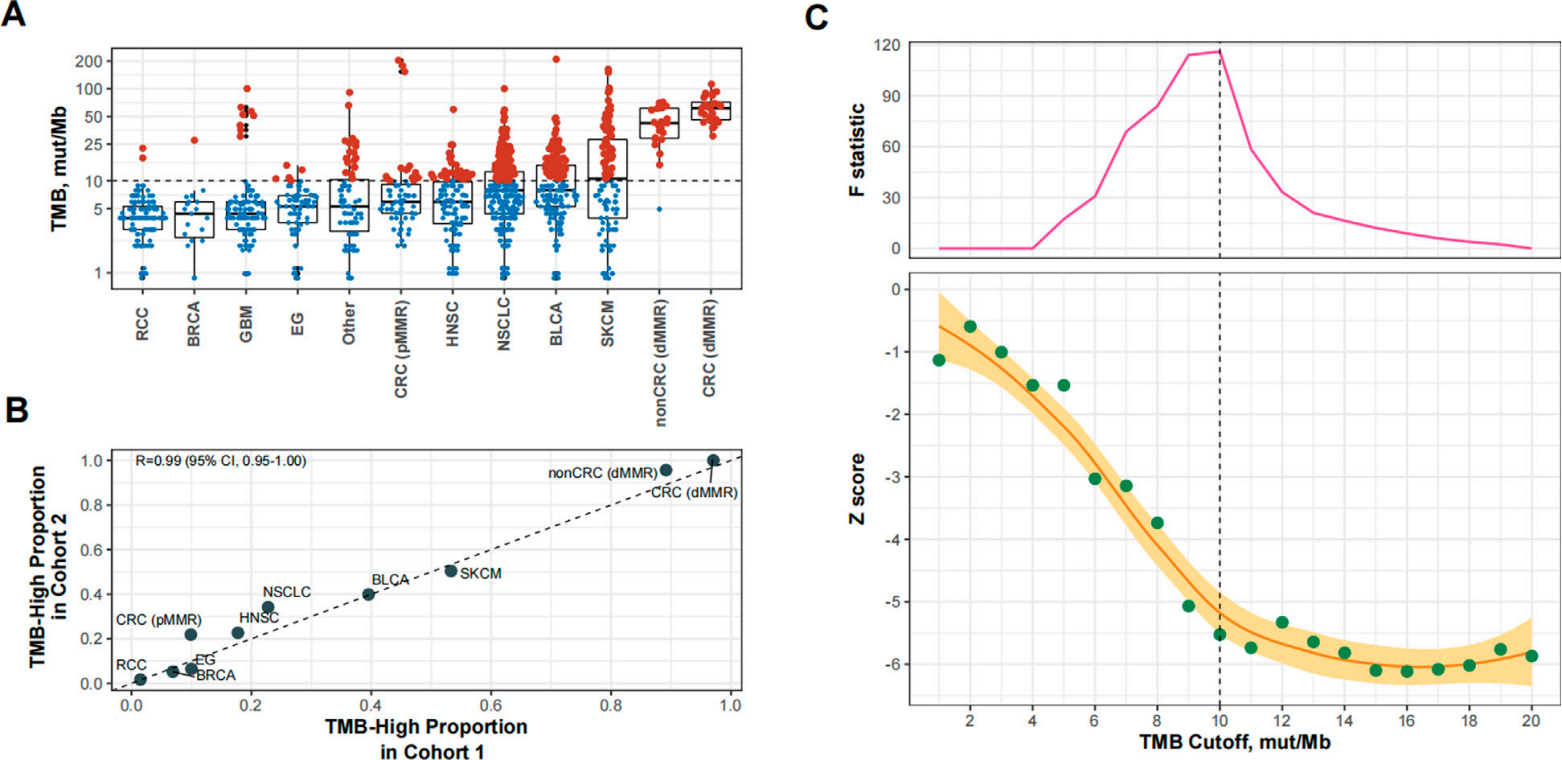
Patient-level analysis to verify the identified tumor mutational burden (TMB) cutoff for defining TMB-high in predicting overall survival benefits of PD-(L) 1 inhibitors. **(A)** TMB-high (i.e., TMB≥10 mut/Mb). cases across cancer types in Cohort 2. Abbreviations: RCC, renal-cell carcinoma; BRCA, breast cancer; GBM, glioma; EG, esophagogastric cancer; HNSC, head and neck cancer; NSCLC, non-small-cell lung cancer; BLCA, bladder cancer; SKCM, melanoma; CRC (dMMR), microsatellite-instable colorectal cancer; CRC (pMMR), microsatellite-stable colorectal cancer; nonCRC (dMMR), microsatellite-instable non-colorectal cancer. **(B)** The proportional agreement between TMB-high cases in Cohorts 1 and 2 when the cutoff was 10 mut/Mb. **(C)** The standardized prognostic impact of TMB (measured by the Wald test Z score, i.e., β coefficient of TMB divided by its standard error in Cox proportional hazards model adjusted for age, sex, panel version, and cancer type), as well as the Chow F statistic, are plotted against the varying cutoffs for TMB. The yellow curve denotes the LOESS smoother. A structural breakpoint at 10 that maximized the Chow F statistic (i.e., minimizing the ordinary least squares estimator) was identified by Chow test.

We then used 10 mut/Mb as the universal cutoff to define TMB-high in Cohort 2 (Supplementary Table S2 and Figure 3A). The proportion of TMB-high cases in this cohort exhibited excellent consistency with Cohort 1 (Figure 3B). Figure 3C shows the relationship between the TMB cutoff and corresponding impact of TMB-high on OS. With a higher cutoff for TMB, the improvement in OS with TMB-high increased and then plateaued, with the structural breakpoint also at 10 mut/Mb (Figure 3C).

**FIGURE 4:**
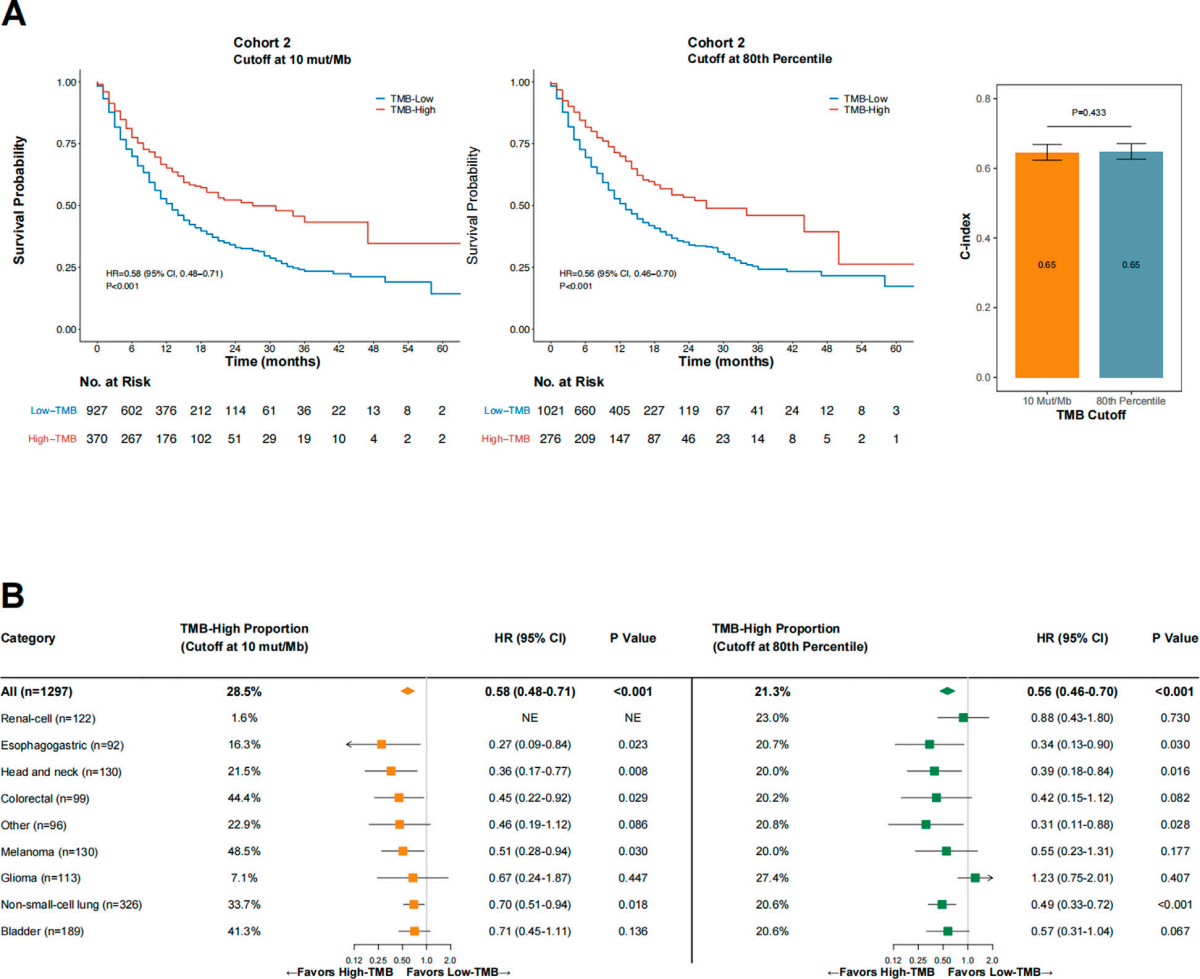
The impact of TMB status (high vs. low) on overall survival in Cohort 2 when the cutoff was 10 mut/Mb **(A)**, left panel) or the 80th percentile per cancer type **(A)**, middle panel), and the comparative concordance indices **(A)**, right panel), as well as subgroup analysis findings across cancer types **(B).** Abbreviations: HR, hazard ratio; CI, confidence interval; C-index, concordance index.

Next, we sought to evaluate the performance of 10 mut/Mb compared with the 80th percentile per cancer type as the cutoff for defining TMB-high. The proportion of TMB-high cases was significantly higher when the cutoff was at 10 mut/Mb than at the 80th percentile per cancer type (28.5% vs. 21.3%, *p* < 0.001). As shown in Figure 4A, the impact of TMB-high on OS was comparable when the cutoff was at 10 mut/Mb (HR = 0.58 [95% CI, 0.48–0.71], *p* < 0.001) or the 80th percentile per cancer type (HR = 0.56 [95% CI, 0.63–0.70], *p* < 0.001). The C-index for TMB-high defined by 10 mut/Mb was similar with that by the 80th percentile per cancer type (*p* = 0.433). Notably, consistent findings were obtained when only microsatellite stable (MSS) cases were analyzed (Supplementary Figure S2).

As shown in Figure 4B, OS was consistently in favor of TMB-high across cancer types when the cutoff was at 10 mut/Mb (test of interaction between TMB status and cancer type, *p* = 0.756). In contrast, TMB-high defined by the 80th percentile per cancer type failed to demonstrate improved OS in patients with glioma and renal-cell carcinoma (test of interaction between TMB status and cancer type, *p* < 0.001). For renal-cell carcinoma, using the 80th percentile as the cutoff provided a markedly higher percentage of TMB-high cases than when using 10 mut/Mb as the cutoff (23.0% vs. 1.6%, *p* < 0.001); however, TMB-high defined by the 80th percentile was not associated with improved OS in this cancer type (HR = 0.88 [95% CI, 0.43–1.80], *p* = 0.730). The two TMB-high renal-cell carcinoma cases defined by the cutoff at 10 mut/Mb both remained alive at the last follow-up (OS duration, 43 and 26 months, respectively). Similar findings were observed in patients with glioma. For melanoma, using 10 mut/Mb as the cutoff identified a higher percentage of TMB-high cases than using the 80th percentile (48.5% vs. 20.0%, *p* < 0.001); TMB-high was associated with significantly improved OS at the former cutoff but not the latter, although the OS HRs were comparable at both cutoff values.

### The reliability of the identified cutoff in defining TMB-high across panels

Based on WES data from 9,821 samples across 32 solid tumors from the TCGA project, we generated *in silico* gene panels that comprised the genes included in the 468-gene MSK-IMPACT, 324-gene F1CDx, or 507-gene PGDx. The percentage of shared genes with MSK-IMPACT was above 70% for both F1CDx and PGDx (80.0% and 74.1%, respectively). When 10 mut/Mb was used as the universal cutoff in defining TMB-high across these panels, the concordance between F1CDx and MSK-IMPACT and between PGDx and MSK-IMPACT were both excellent (Kappa = 0.808 and 0.803, respectively). The concordance between WES and MSK-IMPACT in defining TMB-high was less prominent (Kappa = 0.767) compared with that between F1CDx and MSK-IMPACT and that between PGDx and MSK-IMPACT. One possible explanation is that frequently mutated genes are enriched in gene panels, and hence the average TMB level for WES was significantly lower than that for MSK-IMPACT (mean difference in TMB: −1.71 mut/Mb [95% CI, 1.63–1.78], paired t-test *p* < 0.001). Based on this finding, we further used a lower universal cutoff of 8 mut/Mb for WES and observed an improved concordance (Kappa = 0.814) between the WES-based TMB-high cases (i.e., TMB≥8 mut/Mb) and MSK-IMPACT-based TMB-high cases (i.e., TMB≥10 mut/Mb).

**FIGURE 5:**
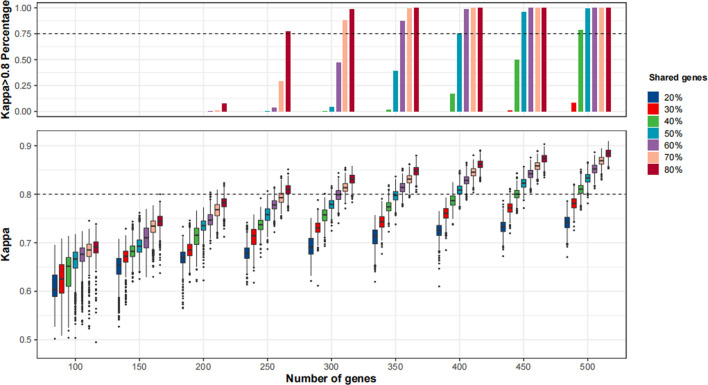
The agreement of tumor mutational burden (TMB)≥10 mut/Mb cases between *in silico* panels comprising genes in MSK-IMPACT and *in silico* panels with various sizes and percentages of shared genes with MSK-IMPACT. 1,000 resampling was performed for each given panel size and percentage of genes shared with MSK-IMPACT. The bioinformatics pipeline for MSK-IMPACT was applied to the randomly sampled panels.

As shown in Figure 5 and Supplementary Figure S3, the agreement of TMB≥10 mut/Mb cases between MSK-IMPACT and the randomly sampled panels was continuously improved with the increase in panel size and percentages of shared genes with MSK-IMPACT. When we applied the MSK-IMPACT pipeline to calculate TMB for the randomly sampled panels, a Kappa>0.80 was achieved in the vast majority (>75%) of the panels when the panel size was at least 250 with at least 80% of genes shared with MSK-IMPACT (Figure 5). When the F1CDx pipeline was applied, a Kappa>0.80 was achieved among the vast majority of the panels when the panel size was at least 350 with at least 80% of genes shared with MSK-IMPACT (Supplementary Figure S3A); when the PGDx pipeline was applied, a Kappa>0.80 was achieved in the vast majority when the panel size was at least 450 with at least 70% of genes shared with MSK-IMPACT (Supplementary Figure S3B).

## Discussion

In this study, we first identified 10 mut/Mb as the optimal universal cutoff for MSK-IMPACT in defining TMB-high across 25 malignancies, on the basis of cancer type-level correlation analysis. We further verified this cutoff as the optimum when defining TMB-high (via MSK-IMPACT) in predicting OS benefits from anti-PD-(L) 1 therapy in a large cohort of patients with advanced cancers. Moreover, *in silico* analyses of large-scale TCGA WES data revealed excellent agreement of the TMB≥10 mut/Mb cases between MSK-IMPACT and F1CDx, between MSK-IMPACT and PGDx, and between MSK-IMPACT and various randomly sampled panels. As such, our findings firmly support the prespecified TMB cutoff in KEYNOTE-158 (Marabelle et al., 2019; Marabelle et al., 2020) and provide rigorous evidence beyond KEYNOTE-158 for the utility of TMB≥10 mut/Mb as a positive indicator of clinical responses and OS benefits from PD-(L) 1 blockade for a broader range of tumors and panels.

Tumor-specific TMB thresholds for prediction of anti-PD-(L) 1 efficacy have not yet been well established; and it would be challenging and labor-intensive to achieve this goal for each cancer type. As the TMB level varies greatly among cancer types (Chalmers et al., 2017; Zehir et al., 2017), it is intuitive to consider that the cutoff to define TMB-high varies by cancer type as well. Likewise, Samstein et al. applied the 80th percentile per cancer type (ranging between 4.4 mut/Mb and 52.2 mut/Mb) as the cutoff in defining TMB-high, which was shown to be predictive of OS benefits from anti-PD-(L) 1 or anti-CTLA4 therapies among various advanced cancers (Samstein et al., 2019). However, using a fixed percentile as the TMB cutoff is clearly contradictory to the fact that ORR for PD-(L) 1 blockade ranges widely from 0% to above 50% across cancer types (Wang et al., 2023). A universal TMB cutoff could therefore be biologically reasonable as the proportion of TMB-high cases defined by this cutoff would vary by cancer type, thus informing the between-cancer variation in tumor immunogenicity and hence the response to anti-PD-(L) 1 therapy (Goodman et al., 2020). As a representative example in Cohort 2, for melanoma, which is among the tumor types with highest ORRs for PD-(L) 1 blockade, the proportion of TMB-high was substantially higher when the cutoff was 10 mut/Mb rather than the 80th percentile (49% vs. 20%), and TMB-high was associated with significantly improved OS at the former cutoff rather than the latter. For the entire Cohort 2, we also demonstrate that using the optimal universal cutoff of 10 mut/Mb showed comparable predictive capacity with the tissue-specific TMB cutoff (80th percentile), but identified a higher proportion of TMB-high cases (29% vs. 21%), which may bring survival prolongation to a larger population with the use of anti-PD-(L) 1 therapy. Moreover, when using the universal 10 mut/Mb cutoff across cancer types, the efficacy of anti-PD-(L) 1 therapy did not significantly differ across cancer types with all the HRs less than 1, further supporting that 10 mut/Mb is a broadly practical TMB cutoff.

MSI-H and TMB-high (i.e., TMB≥10 mut/Mb) are currently the two FDA-approved tumor-agnostic indications for pembrolizumab. Although MSI-H is strongly predictive of anti-PD-(L) 1 response and can be readily utilized in clinical practice due to its dichotomized nature, its clinical impact is limited by a rather low prevalence (<5%) among advanced solid tumors (Hause et al., 2016). Additionally, prior evidence suggests that anti-PD-(L) 1 efficacy in patients with MSI-H tumors was largely attributed to high TMB (Schrock et al., 2019). Notably, our study showed a clinically relevant prevalence of TMB-high (TMB≥10 mut/Mb) based on MSI-IMPACT (16% in Cohorts 1% and 29% in Cohort 2), among whom the clinical benefits from PD-(L) 1 blockade were evident. Together with the findings from KEYNOTE-158, these current data indicate that TMB≥10 mut/Mb could effectively complement MSI-H in identifying candidates for anti-PD-(L) 1 therapy, thereby bringing survival benefits to a larger population (Subbiah et al., 2020).

TMB≥10 mut/Mb may also serve the design of future biomarker-guided clinical trials on anti-PD-(L) 1 therapy. Specifically, it will be of great interest to conduct a biomarker-enrichment basket trial to investigate the efficacy of PD-(L) 1 inhibitors in TMB-high (TMB≥10 mut/Mb) cases of cancer types with minimal or no response to these agents. A recent study reported an ORR of 11% in patients with metastatic colorectal cancer (25/27 MSS, 2/27 undetermined) with TMB≥9 mut/Mb via F1CDx (Meiri et al., 2020), suggesting that it could still be feasible to use TMB-high to identify and enrich patients that benefit from PD-(L) 1 inhibitors even in cancer types with generally poor response to these agents. Furthermore, TMB≥10 mut/Mb may be applied as a stratification factor in future randomized studies of anti-PD-(L)1 therapy, which could in turn verify its utility as a predictive marker for such treatment.

The limitations of this study are as follows. Firstly, this universal TMB cutoff of 10 mut/Mb was identified and verified based on data from patients treated with anti-PD-(L) 1 monotherapy; it may not be extrapolated directly to anti-PD-(L) 1-based combinations and regimens involving other immune checkpoint inhibitors, e.g., anti-CTLA-4 antibodies. As shown in CheckMate-227 for advanced non-small-cell lung cancer, dual blockade of PD-1 and CTLA-4 significantly improved OS compared with chemotherapy in both patients with TMB≥10 mut/Mb and those with TMB<10 mut/Mb via F1CDx (Hellmann et al., 2019). Secondly, this study relied on retrospective analysis of publicly available data and published trials; therefore, inherent biases might exist and the TMB cutoff of 10 mut/Mb should be further testified in future prospective trials. Thirdly, our *in silico* panel analysis did not fully account for the various bioinformatics pipelines for TMB estimation in existing panels; hopefully, this issue will be adequately addressed by efforts from the Friends of Cancer Research TMB Harmonization Project, which seeks to establish a uniform approach in measuring TMB across different panels (Merino et al., 2020). Finally, as it was previously reported that a higher tissue TMB was associated with a higher risk of immune-related adverse events (Bomze et al., 2019), further studies are warranted to investigate the TMB cutoff that optimizes the risk-benefit ratio for anti-PD-(L) 1 therapy.

In summary, our study provides compelling evidence for using 10 mut/Mb as the optimal, universal cutoff for TMB-high that guides the clinical application of anti-PD-(L) 1 therapy in patients with advanced solid tumors. Our findings substantially extend the evidence from KEYNOTE-158 to a broader coverage of settings, which could help to mitigate the challenges faced by the immuno-oncology community in embracing the agnostic approval of TMB-high as a companion diagnostic to pembrolizumab, and more importantly, bring survival prolongation to a larger population.

## Data Availability

The datasets used and analyzed in the study are publicly available. These data can be found in the cBioPortal for Cancer Genomics (http://cbioportal.org/msk-impact), Figure 1 and Supplementary Table S1 of Yarchoan’s study (DOI: 10.1056/NEJMc1713444), Supplemental Data S1 of Samstein’s study (DOI: 10.1038/s41588-018-0312-8), and the UCSC Xena browser (http://xena.ucsc.edu/).
