# Supplementary material for: Universal cutoff for tumor mutational burden in predicting the efficacy of anti-PD-(L)1 therapy for advanced cancers
Source: Front Cell Dev Biol. 2023 May 25;11:1209243. doi: 10.3389/fcell.2023.1209243 (PMC10248461; doi:10.3389/fcell.2023.1209243)
Supplement: Supplementary file 1 [file DataSheet1.PDF]

Supplement

Universal cutoff for tumor mutational burden in predicting the efficacy of anti-PD-(L)1 therapy for advanced cancers

Mo SF, et al.

Figure S1. Flowcharts for the patient inclusion and exclusion procedures at steps 1, 2, and 3.

Abbreviations: TMB, tumor mutational burden; ORR, objective response rate; WES, whole-exome sequencing; TCGA, The Cancer Genome Atlas.

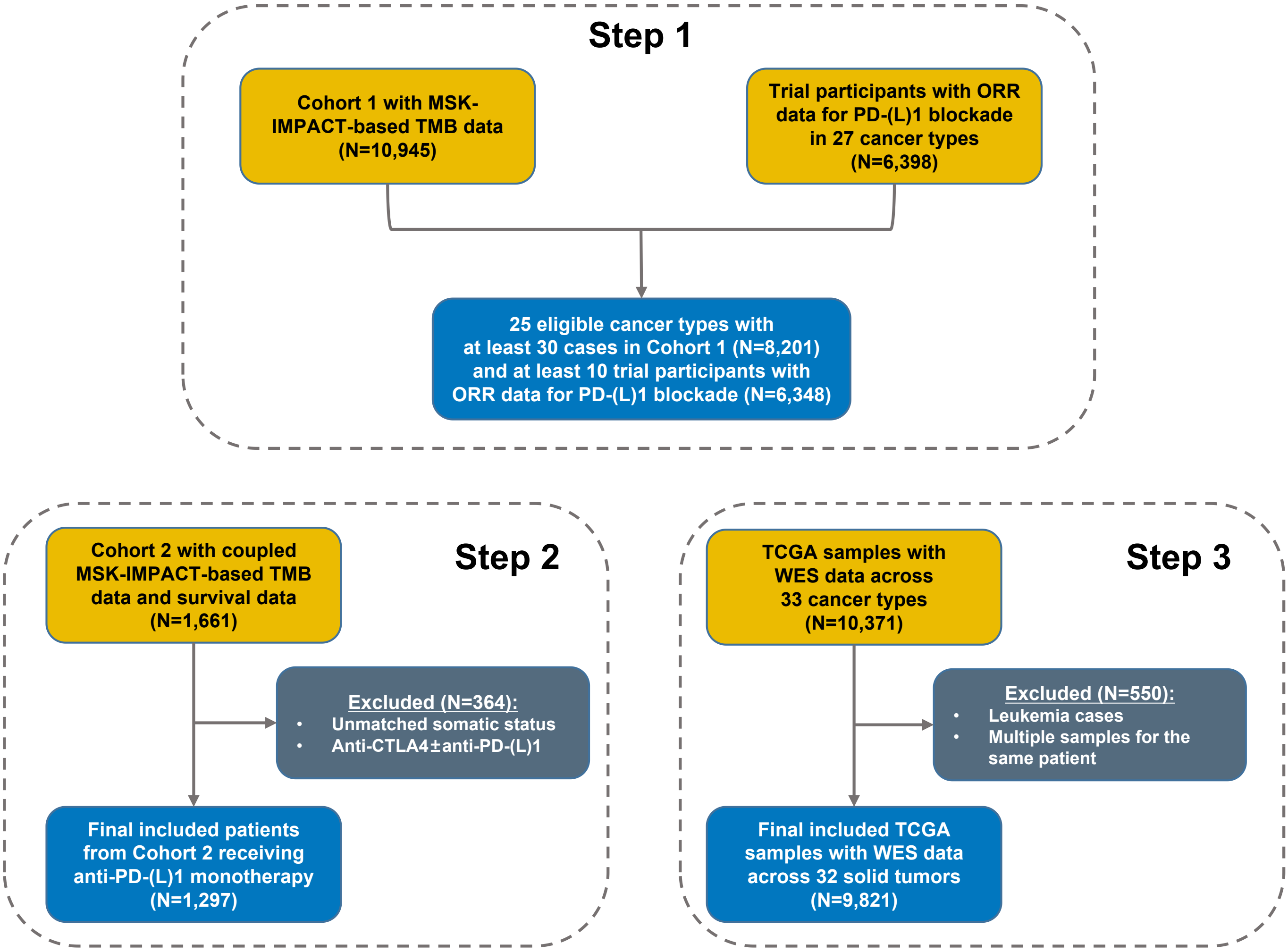

Figure S2. The impact of tumor mutational burden (TMB) status (high vs. low) on overall survival when the cutoff was 10 mut/Mb (left panel) or the 80th percentile per cancer type (right panel) among patients with microsatellite-stable tumors.

Abbreviations: HR, hazard ratio; CI, confidence interval.

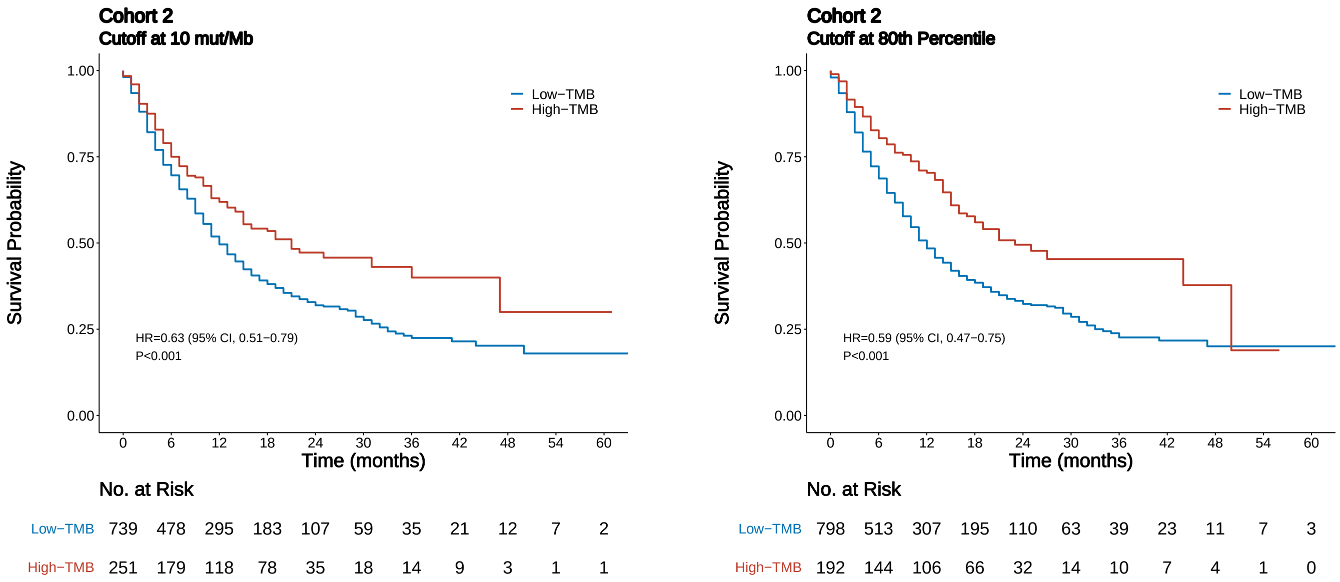

Figure S3. The agreement in tumor mutational burden (TMB)≥10 mut/Mb cases between in silico panels comprising genes in MSK-IMPACT and in silico panels with various sizes and percentages of shared genes with MSK-IMPACT when the bioinformatics pipeline for FoundationOne CDx (A) or PGDx elio tissue complete (B) was applied. 1,000 resampling was performed for each given panel size and percentage of genes shared with MSK-IMPACT.

A

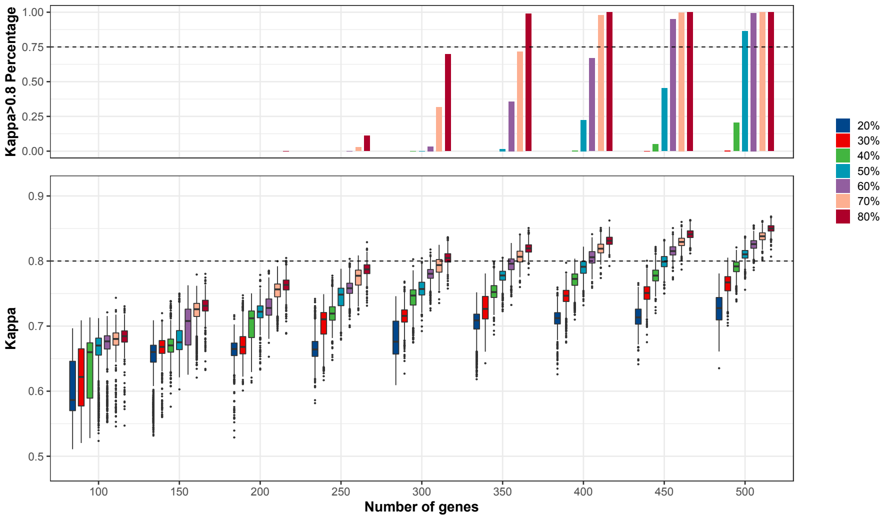

B

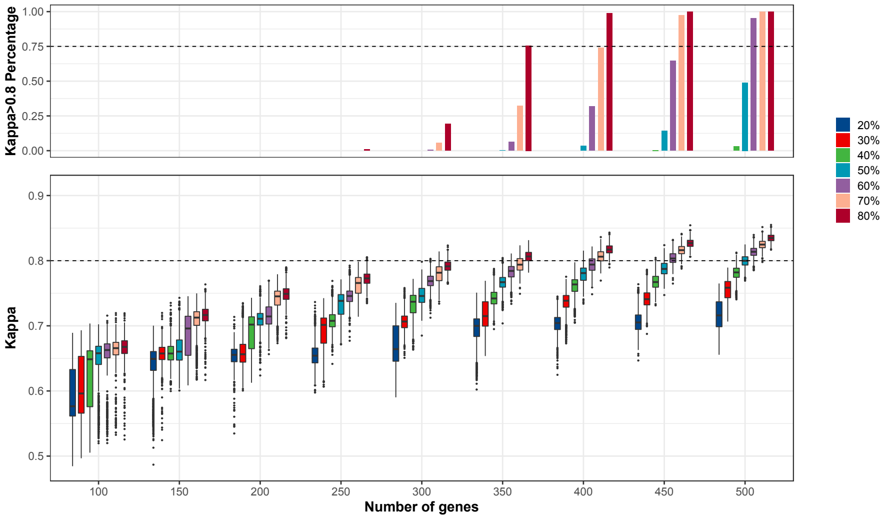

Table S1. The 25 eligible malignancies for cancer-level analysis

Cancer type	No. of Patients in Cohort 1 (N = 8,201)	No. of Patients in trials (N = 6,348)	Objective response rate in trials
Anal carcinoma	32	37	24.3%
Urothelial carcinoma	392	1080	18.1%
Breast carcinoma	1246	280	5.7%
Cervical carcinoma	50	101	14.9%
Microsatellite-instable colorectal cancer	68	135	30.4%
Microsatellite-stable colorectal cancer	879	18	0 %
Cutaneous squamous-cell carcinoma	54	23	52.2%
Esophago-gastric carcinoma	290	678	10.9%
Glioblastoma	282	184	8.7%
Head and neck carcinoma	186	411	14.6%
Renal-cell carcinoma	344	410	25.1%
Hepatocellular carcinoma	85	254	18.1%
Merkel-cell carcinoma	63	25	56.0%
Mesothelioma	87	116	13.8%
Microsatellite-instable non-colorectal cancer	83	77	37.7%
Non-small-cell lung cancer	1505	1080	19.8%
Ovarian cancer	220	161	9.9%
Pancreatic cancer	384	14	0 %
Prostate carcinoma	696	44	6.8%
Sarcoma	618	122	9.0%
Small-cell lung cancer	81	98	11.0%
Skin melanoma	272	917	37.2%
Germ-cell cancer	146	12	0 %
Endometrial carcinoma	95	15	13.3%
Uveal melanoma	43	56	3.6%

Table S2. Baseline characteristics of Cohort 2 (N=1,297)

Variable	N (%)
**Mean age (SD)**	62.34 (13.42)
**Sex**	
Female	478 (36.9)
Male	819 (63.1)
**Cancer type**	
Urothelial carcinoma	182 (14.0)
Breast carcinoma	20 (1.5)
MSI-high colorectal cancer	28 (2.2)
MSS colorectal cancer	65 (5.0)
MSI status undetermined colorectal cancer	6 (0.5)
Esophagogastric carcinoma	82 (6.3)
Glioma	113 (8.7)
Head and neck carcinoma	128 (9.9)
Renal-cell carcinoma	122 (9.4)
MSI-high non-colorectal cancer	23 (1.8)
Non-small-cell lung cancer	325 (25.1)
Melanoma	130 (10.0)
Other	73 (5.6)
**MSK-IMPACT version**	
341-gene	151 (11.6)
410-gene	767 (59.1)
468-gene	379 (29.2)
**MSI status**	
Instable	51 (3.9)
Stable	990 (76.3)
Unknown	256 (19.7)

Abbreviations: MSI, microsatellite instability; MSS, microsatellite stable.
